# Association of metabolic risks with subclinical hypothyroidism: A cross-sectional analysis

**DOI:** 10.12669/pjms.342.13873

**Published:** 2018

**Authors:** Sikandar Hayat Khan, Syed Mohsin Manzoor, Najumusaquib Khan Niazi, Naveed Asif, Aamir Ijaz, Nadeem Fazal

**Affiliations:** 1 Dr. Sikandar Hayat Khan, (FCPS Chemical Pathology). Department of Pathology, PNS Hafeez Hospital, Pakistan; 2 Dr. Syed Mohsin Manzoor, (FCPS Chemical Pathology). Department of Pathology, PNS Hafeez Hospital, Pakistan; 3 Najmusaqib Khan Niazi:, (M.Sc. Healthcare Administration). Healthcare Administration, PNS Hafeez Hospital, Pakistan; 4 Dr. Naveed Asif (FCPS Chemical Pathology). Department of Chemical Pathology & Clinical Endocrinology, Armed Forces Institute of Pathology (AFIP), Rawalpindi, Pakistan; 5 Dr. Aamir Ijaz, (MCPS, FCPS (Chemical Pathology), FRCP, MCPS HPE). Department of Chemical Pathology & Clinical Endocrinology, Armed Forces Institute of Pathology (AFIP), Rawalpindi, Pakistan; 6 Dr. Nadeem Fazal (FCPS Med), Department of Medicine, PNS Hafeez Hospital, Pakistan

**Keywords:** Subclinical hypothyroidism (SCH), HDL-cholesterol, LDL-cholesterol, non-HDL-cholesterol, UACR

## Abstract

**Objective:**

To compare lipid parameters, HbA1c, uric acid and albumin creatinine ratio (UACR) among subjects having euthyroidism, Sub-Clinical Hypothyroidism (SCH) and overt hypothyroidism.

**Methods:**

This comparative cross-sectional analysis was carried out from Dec-2015 to Oct-2016 in collaboration between PNS HAFEEZ hospital and department of chemical pathology and endocrinology, Armed Forces Institute of Pathology, Rawalpindi. Biochemical parameters including lipid indices, HbA1c and UACR were compared between euthyroidism (TSH: 0.5 to 4.0 mIU/L, n=163), subclinical hypothyroidism (TSH: 4.0 to 10 mIU/L, n=16) and overt hypothyroidism (TSH:≥ 10.0 mIU/L, n=9).

**Results:**

LDL-cholesterol, non-HDL-cholesterol and UACR results were as: [(Euthyroid: 2.66 ± 0.73), (SCH: 2.68 ± 0.51) and (Overt hypothyroidism: 3.23 ± 0.59), p-value=0.063], [(Euthyroid: 3.49 ± 0.64), (SCH: 3.35 ± 0.59) and (Overt hypothyroidism: 4.01 ± 0.30), p-value=0.033] and [{Euthyroid: 2.48 (95% CI: 1.63-3.33)}, {SCH: 2.27 (95% CI: 0.37-4.90)} and {Overt hypothyroidism: 14.95 (95% CI: 10.71-19.14){, (p-value< 0.001)] Results for total cholesterol, triglycerides and HDL-cholesterol though increased in overt hypothyroid group were not found to be statistically significant.

**Conclusion:**

LDL-cholesterol, non-HDL-cholesterol and UACR increased from euthyroid subjects to overt hypothyroidism group. However, these changes were found to be more subtle in the subclinical hypothyroid subjects than cases with overt hypothyroidism.

## INTRODUCTION

Cardiovascular diseases (CVD) remain one of the most prevalent causes of morbidity and mortality in the current era. The causes underlying the increased prevalence of CVD have been researched a lot; however, still cases remains where the actual pathogenesis remains obscure.^1^ Overt hypothyroid disease has always been considered to increase CVD related morbidity and mortality.^2^ The diagnosis of hypothyroidism is considered with regards to TSH levels, which in cases of primary thyroid pathology becomes elevated with low T4 and T3. However, in routine clinical practice there are more cases with border line elevations of TSH with normal T4 and T3, where the etiology could not be explained alone by non-thyroidal illnesses (NTI), medications, age or simply a variation of normality.^3^,^4^

Lipid changes in blood especially atherogenic dyslipidemia are considered to actually represent a state of increase atherosclerosis. Thyroid hormones play a significant role in the metabolism of cholesterols by maintaining the normal turnover and utilization at the cellular levels.^5^ While Overt hypothyroidism has been established to dyslipidemia, subclinical hypothyroidism(SCH) in the setting of normal thyroid hormones can also slow down metabolic pathways to accelerate atherosclerotic lipid deposition within vasculature needs more elaboration. Literature review on SCH provides varying conclusions in terms of association with dyslipidemia. Some large sample studies have identified no such association for dyslipidemia in subjects with SCH^6^,^7^, while others suggested the possibility of such an association.^8^ With regards to cardiovascular events, role of lipid metabolism remains instrumental, where the literature review suggests inconsistent evidence. Few studies have found significant dyslipidemia in subjects with SCH^9^,^10^, but studies like Tehran Thyroid Study(TTS) have demonstrated no significant lipid changes in SCH.^11^

Consequent upon these highlighted discrepancies in data we planned to compare various lipid parameters, HbA1c and uric acid and albumin creatinine ratio among subjects with euthyroidism, SCH and overt hypothyroidism.

## METHODS

Our study was a comparative cross-sectional analysis which was carried out at the departments of pathology and medicine PNS HAFEEZ in liaison with department of chemical pathology and endocrinology, Armed Forces Institute of Pathology (AFIP), Rawalpindi. Based upon non-probability convenience sampling, asymptomatic subjects from medical OPDs were requested to participate in the study. Subjects who volunteered were explained about study requirements and consequences, formally consented, interviewed for presence of any chronic metabolic or other disease, clinically examined for measurements of their anthropometric data, and sampled for 10 ml of blood in various phlebotomy tubes. Subjects who had pregnancy, acute infectious disease process, diabetes, IHD and inappropriate medical fasting were excluded from the study. A total sample considered after these exclusions was 188 subjects.

Total cholesterol, triglycerides, LDL-cholesterol and HDL-cholesterol were analyzed using CHOD-PAP, GPO-PAP, cholesterol esterase end-point methods on clinical chemistry analyzer (Selectra-ProM and AVIDA-1800). Serum TSH was measured using chemiluminescence’s technique on Immulite® 1000. Total T4 and Total T3 were measured by competitive ELISA (Human). In case of a TSH result swaying away from 0.5-3.5 mIU/L, free T4 was also measured using chemiluminescence’s technique on Immulite® 1000. HbA1c was measured by fast ion-exchange resin separation method, while UACR thru immune-turbidimetric method on ADVIA-1800 Chemistry System.

Based upon the TSH results subjects were classified as having euthyroidism (TSH: 0.5 to 4.0 mIU/L), subclinical hypothyroidism (TSH: 4.0 to 10 mIU/L) and overt hypothyroidism (TSH: ≥ 10.0 mIU/L).

### Data analysis

All data were entered into SPSS version-15. Descriptive statistics in terms of mean + SD were calculated for various parameters for gender differences. 163 subjects were diagnosed to have euthyroidism, while SCH and overt hypothyroidism were labeled in 16 and 9 subjects. Age, lipid indices, HbA1c, UACR and uric acid were compared between the 3 groups by univariate GLM model as the latter 2 groups had sample size <30.

## RESULTS

**Fig.1:**
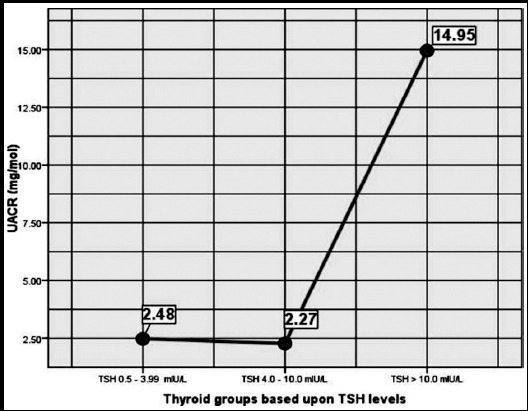
Univariate GLM model with thyroid groups as independent factor and Urine Albumin Creatinine Ratio (UACR) as dependent variable [Euthyroid: 2.48 (95% CI, 1.63-3.33], [SCH: 2.27(95% CI: 0.37-4.90] and [Overt hypothyroidism: 14.95 (95% CI: 10.71-19.14)], (p-value<0.001).

There were 96 males and 92 females in our study. Differences between age, anthropometric indices and biochemical parameters between genders are shown in Table-I. The comparison between age, anthropometric indices, ALT, uric acid and glycated hemoglobin among euthyroid subjects, patients with SCH and overt hypothyroidism is depicted in Table-II. LDL-cholesterol and Non-HDL-cholesterol shows a progressive worsening from euthyroid subjects to subjects having overt hypothyroidism (Table-III). Pearson’s correlation was found to be significant only for LDL-cholesterol and urine albumin creatinine ratio (Table-IV). UACR provided being having an overall significant model p-value depicted minimal change between euthyroid and SCH group (Fig.1).

**Table-I:** Gender differences for various evaluated parameters.

Dependent Variable	Gender	N	Mean	95% Confidence Interval		(p-value)*
				Lower Bound	Upper Bound	
Age (years)	Male	96	46.87	44.06	49.68	0.045
	Females	92	42.91	40.25	45.56	
Weight (Kg)	Male	96	72.66	69.42	75.90	0.460
	Females	92	70.98	67.92	74.05	
Height (cm)	Male	96	169.08	167.32	170.84	< 0.001
	Females	92	158.24	156.58	159.91	
Waist (cm)	Male	96	90.83	88.41	93.26	0.061
	Females	92	94.03	91.74	96.32	
Hip (cm)	Male	96	97.47	95.17	99.78	0.030
	Females	92	101.00	98.82	103.18	
Total cholesterol (mmol/L)	Male	95	4.42	4.29	4.555	0.861
	Females	92	4.44	4.31	4.564	
Fasting triglycerides (mmol/L)	Male	95	1.59	1.43	1.75	0.308
	Females	92	1.48	1.32	1.63	
HDL-cholesterol (mmol/L)	Male	95	0.92	0.86	0.98	0.005
	Females	91	1.04	0.98	1.10	
LDL-cholesterol (mmol/L)	Male	95	2.67	2.50	2.84	0.716
	Females	92	2.63	2.46	2.79	
Non-HDL-cholesterol (mmol/L)	Male	95	3.49	3.34	3.63	0.439
	Females	92	3.41	3.28	3.54	
Uric acid (umol/L)	Male	96	342.01	325.04	359.05	<0.001
	Females	92	276.12	260.05	292.19	
A1c (%)**	Male	95	5.38	5.18	5.58	<0.001
	Females	90	5.84	5.65	6.03	
ALT (IU/L)	Male	96	32.78	26.49	39.07	0.088
	Females	92	25.25	19.31	31.20	
UACR***(mg/mmol)	Male	68	2.50	1.21	3.78	0.393
	Females	75	3.26	2.50	4.48	

* Independent sample t-test,

** HbA1c (Glycated hemoglobin),

*** Urine Albumin Creatinine Ratio(UACR).

**Table-II:** Comparison of various biochemical and anthropometric indices among subjects having euthyroidism, SCH and overt hypothyroidism.

Dependent Variable	Thyroid groups	N	Mean ± SD*	Sig (p-value)
Age (Years)	TSH 0.5 - 3.99 mIU/L	163	45.52 ± 11.50	0.410
	TSH 4.0 - 10.0 mIU/L	16	42.38 ± 9.16	
	TSH > 10.0 mIU/L	9	48.56 ± 15.72	
BMI	TSH 0.5 - 3.99 mIU/L	163	26.93 ± 5.31	0.070
	TSH 4.0 - 10.0 mIU/L	16	30.09 ± 5.22	
	TSH > 10.0 mIU/L	9	26.63 ± 3.72	
Waist to hip ratio (WHpR)	TSH 0.5 - 3.99 mIU/L	163	0.93 ± 0.092	0.619
	TSH 4.0 - 10.0 mIU/L	16	0.94 ± 0.044	
	TSH > 10.0 mIU/L	9	0.95 ± 0.050	
Waist to height ratio (WHtR)	TSH 0.5 - 3.99 mIU/L	163	0.57 ± 0.071	0.120
	TSH 4.0 - 10.0 mIU/L	16	0.61 ± 0.068	
	TSH > 10.0 mIU/L	9	0.58 ± 0.072	
ALT (IU/L)	TSH 0.5 - 3.99 mIU/L	163	29.61 ± 25.87	0.705
	TSH 4.0 - 10.0 mIU/L	16	24.19 ± 12.68	
	TSH > 10.0 mIU/L	9	30.00 ± 22.16	
UA (umol/L)	TSH 0.5 - 3.99 mIU/L	163	304.17 ± 80.08	0.456
	TSH 4.0 - 10.0 mIU/L	16	279.00 ± 72.03	
	TSH > 10.0 mIU/L	9	293.56 ± 61.94	
A1c (%)	TSH 0.5 - 3.99 mIU/L	160	5.73 ± 0.95	0.508
	TSH 4.0 - 10.0 mIU/L	16	5.31 ± 1.37	
	TSH > 10.0 mIU/L	9	5.93 ± 1.12	

* Mean + standard deviation as calculated by univariate general linear model,

** HbA1c (Glycated hemoglobin).

**Table-III:** Comparison of various biochemical and anthropometric indices among subjects having euthyroidism, SCH and overt hypothyroidism.

Dependent Variable	Thyroid groups	n	Mean ± SD	Sig (p-value)*
Total cholesterol (mmol/L)	TSH 0.5 - 3.99 mIU/L	162	4.47 ± 0.59	0.068
	TSH 4.0 - 10.0 mIU/L	16	4.36 ± 0.63	
	TSH > 10.0 mIU/L	9	4.90 ± 0.33	
Triglycerides (mmol/L)	TSH 0.5 - 3.99 mIU/L	162	1.57 ± 0.72	0.113
	TSH 4.0 - 10.0 mIU/L	16	1.36 + 0.46	
	TSH > 10.0 mIU/L	9	1.97 ± 0.58	
HDL-cholesterol (mmol/L)	TSH 0.5 - 3.99 mIU/L	162	0.98 ± 0.27	0.347
	TSH 4.0 - 10.0 mIU/L	16	1.02 ± 0.19	
	TSH > 10.0 mIU/L	9	0.86 ± 0.15	
LDL-cholesterol (mmol/L)	TSH 0.5 - 3.99 mIU/L	162	2.66 ± 0.73	0.063
	TSH 4.0 - 10.0 mIU/L	16	2.68 ± 0.51	
	TSH > 10.0 mIU/L	9	3.23 ± 0.59	
Non- HDL-cholesterol (mmol/L)	TSH 0.5 - 3.99 mIU/L	162	3.49 ± 0.64	0.033
	TSH 4.0 - 10.0 mIU/L	16	3.35 ± 0.59	
	TSH > 10.0 mIU/L	9	4.01 ± 0.30	

* Mean + standard deviation as calculated by univariate general linear model.

**Table-IV:** Person’s correlation between TSH levels and lipid parameters, UACR, A1c and uric acid.

Parameter	N	Pearson’s correlation Coefficient (r)	Sig 2-tailed (p-value)
Age (Years)	188	0.049	0.503
Total cholesterol (mmol/L)	187	0.137	0.062
Fasting triglycerides (mmol/L)	187	0.049	0.504
HDL-cholesterol (mmol/L)	187	-0.031	186
LDL-cholesterol (mmol/L)	187	0.220	0.003
Non- HDL-cholesterol (mmol/L)	187	0.134	0.066
Uric acid (umol/L)	188	-0.005	0.949
Urine albumin creatinine ration (mg/mol)	143	0.374	<0.001
A1c (%)	185	0.108	0.145
ALT (IU/L)	188	-0.003	0.972

## DISCUSSION

Our study has shown a worsening pattern for lipid indices from euthyroidism to overt hypothyroidism. However, this pattern was not found to be more tangible in the SCH group except for LDL and non-HDL cholesterol. These results are consistent with the findings of Laway et al., Turham et al., Dunts et al. and the Tromso Study.^12^-^15^ While the Tehran Lipid and Glucose Study (TLGS) and some other researchers did not find significant differences among subjects within SCH and control group.^16^-^19^

The first question from our data arises as to why the SCH group did not show marked changes in lipids as compared to the overt hypothyroid group. The first possibility could be the graded decline of primary thyroid functioning which at this stage of SCH may not be associated with metabolic complications. Secondly, considering primary thyroid abnormality to initiate metabolic derangements in the presence of normal pancreatic and hepatocyte function would allow compensatory mechanisms to reduce metabolic derangements at this stage. The evidence for this observation comes from the compensatory hyper-stimulation in insulin sensitivity which has been demonstrated by certain researchers.^20^,^21^ This compensatory insulin release could in theory result in slight improvement in lipid parameters like triglycerides and HDL-cholesterol as we have observed in our work. But our study did find increase in LDL and non-HDL-cholesterol which may predict an early decompensation and possible risks for cardiovascular disease. Lastly, it must also be taken into consideration that TSH cut-offs as being employed to diagnose SCH have been varying across literature with studies using a higher TSH cut-offs like 15 mIU/L have demonstrated higher dyslipidemias.^14^,^22^ Staub et al. have found worsening of various inflammatory markers including LDL-cholesterol in subjects with TSH greater than12 mIU/L.^23^

### Limitations

Firstly, our study remains inclusive of sub-continental subjects who are otherwise considered to be more prone towards metabolic risk,^24^ highlighting a multi-factorial risk assessment for CVD risk calculation. Secondly, we do acknowledge the possibility of Type-2 statistical error resulting from decreased sample size. The matter could not be addressed as the sampling was made randomly and multiple exclusions were made due to patients already suffering from IHD, hypertension, diabetes or other chronic disorder. Moreover, the study design being a cross-sectional one was not designed to establish a cause to effect ratio for which we recommend a more appropriate randomized controlled trial.

### Clinically important

Our study is clinically important because it indirectly emphasized the importance of the subclinical hypothyroidism where every attempt should be made to find metabolic risks. Also the cross-sectional study provides new avenues for doing randomized controlled trials to further segregate multi-risk clustered Pakistani population considering worsening of thyroid function.

## CONCLUSION

Lipid parameters were observed to get deteriorated from euthyroid subjects to patients having overt hypothyroidism. However, these changes were found to be more subtle in the subclinical hypothyroid group than cases with overt hypothyroidism. Out of all evaluated parameters LDL-cholesterol, non-HDL-cholesterol and urine albumin creatinine ratio demonstrated the most significant differences.

### Authors’ contribution

***Sikandar Hayat Khan*** Study design, data collection, analysis, results and discussion.

***Syed Mohsin Manzoor and Nadeem Fazal*** Data collection, analysis.

***Najmusaqib Khan Niazi*** Data collection, analysis, article design.

***Naveed Asif*** Results, analysis and discussion.

***Aamir Ijaz*** Study design, discussion.

***Sikandar Hayat Khan*** takes the responsibility and is accountable for all aspects of the work in ensuring that questions related to the accuracy or integrity of any part of the work are appropriately investigated and resolved.
